# Nonlinear Hall Effect in a Semiconductor Heterostructure with Rashba and Dresselhaus Effects

**DOI:** 10.1021/acs.nanolett.6c03601

**Published:** 2026-11-02

**Authors:** Jeong Ung Ahn, Junyeong Choi, Minseong Kwon, Won-Young Choi, Ki Hyuk Han, Cheol Jun Kim, Chaun Jang, Dong-Soo Han, OukJae Lee, Byoung-Chul Min, Kyoung-Whan Kim, Hyun Cheol Koo

**Affiliations:** † KU-KIST Graduate School of Converging Science and Technology, 34973 Korea University, Seoul 02841, Republic of Korea; ‡ Center for Semiconductor Technology, 58975 Korea Institute of Science and Technology, Seoul 02792, Republic of Korea; § Department of Physics, 26721 Yonsei University, Seoul 03722, Republic of Korea; ∥ Center for Quantum Dynamics of Angular Momentum, Pohang University of Science and Technology, Pohang 37673, Republic of Korea; ⊥ Department of Physics, Kyung Hee University, Seoul 02447, Republic of Korea; # Department of Materials Science and Engineering, Seoul National University, Seoul 08826, Republic of Korea

**Keywords:** nonlinear Hall effect, two-dimensional electron gas, spin−orbit coupling, Rashba effect, Dresselhaus effect, skew-scattering

## Abstract

We report a nonlinear Hall effect in a high-mobility InAs quantum-well two-dimensional electron gas (2DEG) with coexisting Rashba and Dresselhaus spin–orbit interactions. Under zero magnetic field, a second-harmonic transverse electric field emerges that scales quadratically with current density and is significantly larger than the second-harmonic longitudinal response. Angle-dependent measurements reveal a pronounced 2-fold anisotropy that follows the combined spin–orbit field. This 2-fold symmetry reflects the anisotropic spin–orbit field generated by coexisting Rashba and Dresselhaus interactions rather than crystal symmetry. The normalized signal reaches ∼10^–9^ V^–1^ m and persists up to room temperature. Conductivity scaling shows a quadratic dependence consistent with skew-scattering-dominated transport. These results establish conventional semiconductor 2DEGs as a promising platform for spin-orbit-driven nonlinear transport and rectification functionalities.

## Graphical abstract

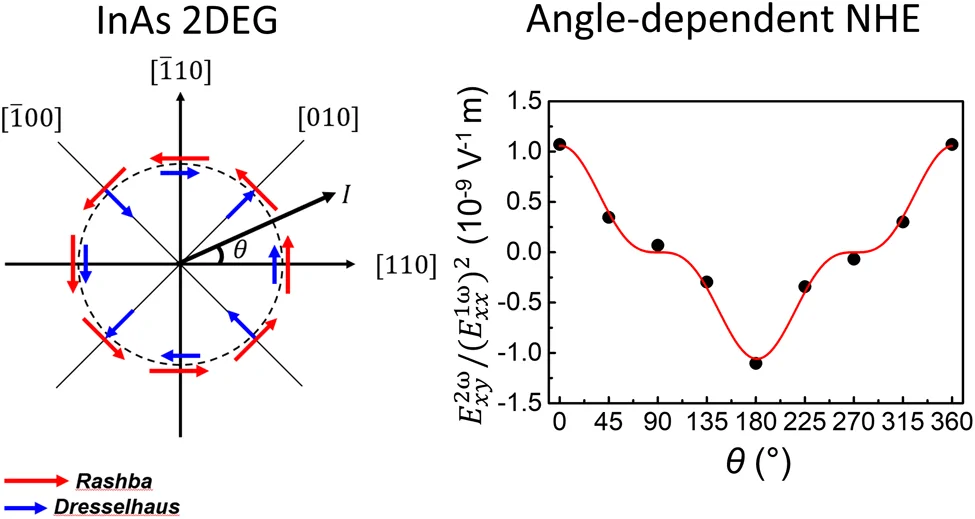

The Hall effect and its anomalous counterpart have long served as central probes of symmetry breaking and band topology in solids, typically requiring time-reversal symmetry breaking induced by magnetic order or an external magnetic field.^1–3^ Recent theoretical studies have shown that even in nonmagnetic conductors, inversion symmetry breaking can generate a second-order transverse response through the Berry-curvature dipole (BCD), giving rise to the nonlinear Hall effect (NHE), in which the transverse current scales quadratically with the applied electric field.^4–6^ This mechanism has attracted growing interest for both its fundamental implications and as a route to nonlinear rectification, frequency doubling, and terahertz photodetection.^7–10^

Two complementary microscopic mechanisms for the NHE have been identified. An intrinsic contribution arises from the BCD,^11^ whereas extrinsic processes, including skew scattering and side-jump scattering, can also generate nonlinear Hall responses with characteristic conductivity scaling.^12,13^ Experimentally, sizable nonlinear Hall signals have been established primarily in noncentrosymmetric and topological quantum materials, including low-symmetry transition-metal dichalcogenide semimetals,^7,8^ Weyl or Dirac semimetals,^11,13^ topological and crystalline topological insulators,^9,14^ Rashba spin–orbit-coupled polar semiconductors,^15^ and oxide-interface two-dimensional electron gases.^16^

Despite these advances, nonlinear Hall effects have thus far been realized primarily in exotic quantum materials or engineered interfaces, rather than in conventional semiconductor heterostructures. Whether comparable nonlinear responses can arise in a III–V semiconductor quantum-well two-dimensional electron gas (2DEG) has remained an open question. Semiconductor 2DEGs provide an appealing and technologically compatible platform because spin–orbit coupling naturally induces effective inversion asymmetry through Rashba and Dresselhaus interactions while preserving high mobility and excellent electrostatic tunability.^17–21^ However, NHEs in such Rashba-type semiconductor systems have not been systematically established.

We investigate nonlinear Hall transport in a high-mobility InAs quantum-well 2DEG hosting coexisting Rashba and Dresselhaus interactions.^21^ Under zero magnetic field, a second-harmonic transverse electric field is observed that exceeds the second-harmonic longitudinal component and exhibits a clear quadratic dependence on current density. Angle-resolved measurements reveal a 2-fold crystallographic anisotropy consistent with the vectorial combination of Rashba and Dresselhaus spin–orbit fields. Temperature- and conductivity-dependent analyses further indicate scaling behavior characteristic of skew-scattering-dominated mechanisms, and the nonlinear response persists up to room temperature.

This work establishes a technologically compatible semiconductor platform in which anisotropic spin–orbit coupling can generate nonlinear transverse transport without relying on topological or Weyl band structures. More broadly, the present study highlights the potential of conventional III–V semiconductor heterostructures for exploring nonlinear Hall phenomena and their microscopic origins. Such a system may provide opportunities for investigating intrinsic and extrinsic mechanisms of nonlinear Hall transport while suggesting routes toward room-temperature nonlinear rectification and spin-orbit-engineered functionalities in conventional semiconductor devices.

To investigate how anisotropic spin–orbit coupling influences nonlinear Hall transport in a conventional semiconductor, we fabricated Hall-bar devices based on a high-mobility InAs quantum-well 2DEG. The coexistence of Rashba and Dresselhaus interactions provides a model platform for examining the relationship between crystallographic anisotropy and nonlinear transverse transport in a conventional semiconductor environment. We first establish the basic transport characteristics of the device and verify the nonlinear origin of the observed second-harmonic transverse response before examining its angular and temperature dependences.

Figure 1a shows an InAs-based 2DEG heterostructure used in this study. The built-in structural asymmetry of the heterostructure generates an internal electric field perpendicular to the 2DEG plane, which induces a sizable Rashba-like spin–orbit interaction. Figure 1b illustrates the Hall-bar device geometry used for nonlinear Hall measurements, where the longitudinal and transverse voltages were simultaneously measured under an ac current applied along the channel. Figure 1c presents the first-harmonic electric field measured along the longitudinal (*E*_*xx*_^1ω^) and transverse (*E*_*xy*_^1ω^) directions of the channel. *E*_*xx*_^1ω^ exhibits a linear dependence on the applied ac current, confirming that charge transport remains in the ohmic regime under the measurement conditions.

**Figure 1:**
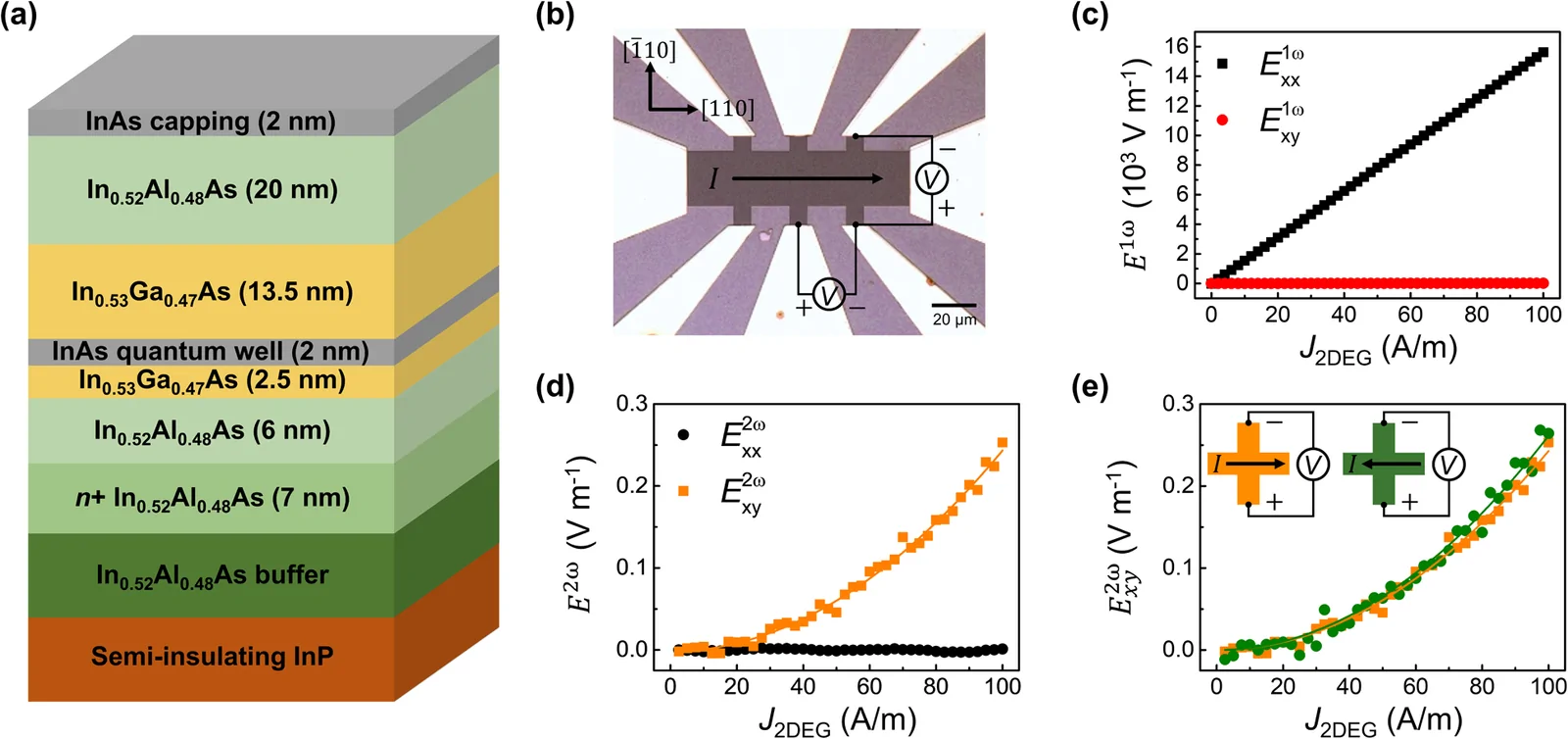
Device structure and representative nonlinear Hall response of the InAs quantum well. (a) Schematic of the InAs quantum well heterostructure. (b) Optical microscopy image of Hall-bar device. (c) First-harmonic and (d) second-harmonic longitudinal and transverse electric fields as a function of current density. (e) Second-harmonic transverse electric field measured for opposite current directions. The solid lines are quadratic fits to the data. The data in panels (c)–(e) were measured at 300 K with the ac current applied along the [110] axis.

In contrast, *E*_*xy*_^1ω^ is significantly smaller than *E*_*xx*_^1ω^. The residual transverse response is attributed to a minor mixing of the longitudinal signal, which can arise from slight probe misalignment and intrinsic resistance anisotropy of the channel. As shown in Figure 1d, the second-harmonic longitudinal electric field *E*_*xx*_^2ω^ is much smaller than the transverse field *E*_*xy*_^2ω^ over the entire current density range. This pronounced dominance of the transverse nonlinear response is inconsistent with trivial thermal effects such as Joule heating, which would primarily manifest in the longitudinal channel. Figure 1e shows *E*_*xy*_^2ω^ as a function of 2DEG current density *J*_2DEG_. The magnitude of *E*_*xy*_^2ω^ follows a parabolic dependence on the applied current, consistent with a second-order nonlinear transport response. Notably, the sign of the second-harmonic transverse voltage remains unchanged upon reversing the current direction, indicating that the observed signal is even in current.

At room temperature, we perform angle-dependent nonlinear Hall effect measurements by rotating the in-plane current direction relative to the crystallographic axes. Figure 2a illustrates the measurement geometry, where the current angle θ is defined with respect to the [110] crystallographic direction. This approach allows direct probing of how crystal symmetry influences both linear and nonlinear transport responses. Figure 2b and 2c show *E*_*xx*_^1ω^ and *E*_*xy*_^1ω^, respectively, measured as a function of current density for representative current angles of 0°, 45°, 90°, and 135°. For all orientations, *E*_*xx*_^1ω^ increases linearly with current density, confirming ohmic transport. However, the slope exhibits a clear angular dependence: *E*_*xx*_^1ω^ is largest at θ = 90° and smallest at θ = 0°, while the signals at θ = 45° and 135° show nearly identical magnitudes. *E*_*xy*_^1ω^ remains much smaller than *E*_*xx*_^1ω^ and reverses sign between 45° and 135°.

**Figure 2:**
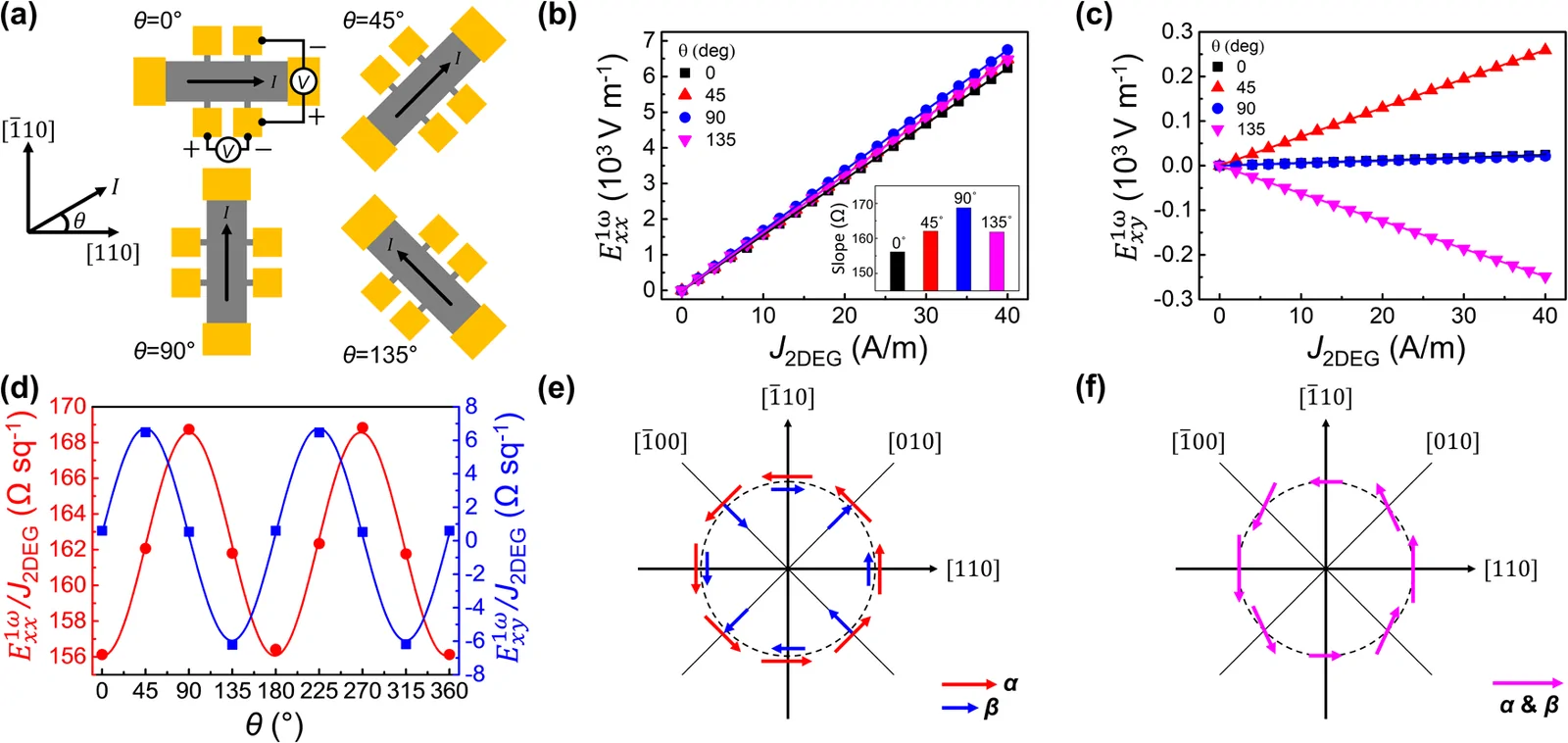
Angle-dependent linear transport. (a) Measurement geometry defining the in-plane current angle θ with respect to [110]. First-harmonic (b) longitudinal and (c) transverse electric fields for representative current angles (solid lines: linear fits). The inset in panel (b) summarizes the slope values extracted from the linear fits for each current direction. (d) Extracted longitudinal and transverse sheet resistances (solid lines: cos 2θ and sin 2θ fits). Schematics of (e) Rashba and Dresselhaus spin-orbit fields and (f) total spin-orbit field. The arrows represent momentum-dependent effective fields in **k**-space. All measurements were performed at 300 K.

Figure 2d summarizes the angular dependence of the longitudinal and transverse sheet resistances extracted from the slopes of the linear fits to the first-harmonic data in Figure 2b and 2c. The longitudinal component exhibits a clear 2-fold angular dependence that follows a −cos 2θ form, while the transverse component varies as sin 2θ with an approximately 45° phase shift relative to the longitudinal response, indicating an in-plane anisotropy of the linear transport. The 2θ dependence is not expected for a pure Rashba interaction, which is rotationally symmetric about the *z*-axis, but is consistent with the coexistence of Rashba and Dresselhaus interactions, the latter arising from bulk inversion asymmetry.^20^ Figures 2e and 2f schematically illustrate the spin–orbit fields generated by the Rashba and Dresselhaus interactions in the InAs quantum well. When both interactions coexist, the total spin–orbit field **d**(**k**) [see eq 2] becomes anisotropic, with |d(k)| = $\left|k\right|\sqrt{\alpha^{2}+\beta^{2}+2\alpha \beta \;\cos2\gamma }$ , where α and β are the Rashba and Dresselhaus coefficients, respectively, and γ is the angle of the crystal momentum **k** with respect to the [110] direction.^21^ This 2-fold modulation of the effective spin–orbit field produces anisotropic band dispersion and Fermi-surface distortion, which can naturally give rise to the cos 2θ and sin 2θ variations.^22^ These considerations suggest that the coexistence of Rashba and Dresselhaus spin–orbit interactions plays an important role in the observed 2-fold anisotropy of the first-harmonic response.

To substantiate this interpretation, we formulate a transport model based on the steady-state quantum Liouville equation:

1 $$\frac{i}{\hbar }\left[H_{k},\rho_{k}\right]-\frac{eE}{\hbar }\cdot D_{k}\rho_{k}={\left(\partial_{t}\rho_{k}\right)}_{\mathrm{sc}}$$

where ρ_**k**_ is the electronic density matrix, *e* > 0 is the elementary charge, and **E** is the applied electric field. The Hamiltonian

2 $$H_{k}=\frac{\hbar^{2}k^{2}}{2m^{*}}+\alpha \left(\sigma_{x}k_{y}-\sigma_{y}k_{x}\right)+\beta \left(\sigma_{x}k_{x}-\sigma_{y}k_{y}\right)\equiv \frac{\hbar^{2}k^{2}}{2m^{*}}+d\left(k\right)\cdot \sigma$$

describes a Rashba-Dresselhaus (RD) 2DEG, where α and β are the Rashba and Dresselhaus coefficients, respectively, m* is the effective mass, **σ** denotes the Pauli matrices, and *k* = |**k**|. The covariant derivative $D_{k}$ is defined by ${\left[D_{k}B_{k}\right]}_{nm}$ = $\nabla_{k}B_{knm}-i{\left[A\left(k\right),B_{k}\right]}_{nm}$ , where **A**_*nm*_ (**k**) = *i*⟨*n* **k**|∇_**k**_|*m* **k**⟩ is the Berry connection matrix, |*n* **k**⟩ denotes the eigenstates of *H*_**k**_, and *n*,*m* = ± are the spin-band indices. For simplicity, we treat the scattering term within the relaxation-time approximation, ${\left(\partial_{t}\rho_{k}\right)}_{\mathrm{sc}}=-\frac{\rho_{k}-\rho_{k}^{\left(0\right)}}{\tau }$ , where ρ_**k**_^(0)^ = ∑_*n*_ *f*_*n*__**k**_ |*n* **k**⟩⟨*n* **k**| is the equilibrium density matrix, *f*_*n*__**k**_ is the Fermi–Dirac distribution, and τ is the relaxation time. The 2-fold anisotropy of the model is encoded in the energy eigenvalues $\frac{\hbar^{2}k^{2}}{2m^{*}}\pm \left|d\left(k\right)\right|$ and the eigenstates |*n* **k**⟩ of *H*_**k**_. The constant relaxation-time approximation provides a widely used effective treatment of scattering from short-range impurities, consistent with the short-range impurity model used below for the second-order response. Although the use of the relaxation-time approximation limits the quantitative predictive power of the Rashba–Dresselhaus model,^23–25^ we adopt it here because it provides a simple and transparent framework for capturing the qualitative mechanism and characteristic angular dependence, rather than for quantitatively predicting the absolute magnitude of the response.

To calculate the response to the applied electric field, we obtain the first-order change ρ_**k**_^(1)^ by evaluating the electric-field term in eq 1 with ρ_**k**_^(0)^. The first-harmonic current is then calculated as $j_{i}^{\left(1\right)}$ = $-\frac{e}{S}\underset{k}{\sum }\mathrm{Tr}\left[v_{ki}\rho_{k}^{\left(1\right)}\right]$ , where $v_{ki}=\frac{1}{\hbar }\partial_{k_{i}}H_{k}$ is the velocity operator along the *i*-direction and *S* is the area of the system. To obtain analytic expressions, we assume *ℏ*/τ ≪ 2|**d**(**k**)| ≪ *E*_*F*_, where 2|**d**(**k**)| is the RD spin–orbit splitting and *E*_*F*_ is the Fermi energy. The first inequality corresponds to the clean limit, while the second allows us to treat the spin–orbit splitting as small (compared to the Fermi energy). Following the procedure in Supplementary Note 1 in the Supporting Information, we obtain the linear electrical conductivity tensor σ_*ij*_ defined by *j*_*i*_^(1)^ = ∑_*j*_σ_*ij*_ *E*_*j*_ as

3a $$\sigma_{xx}=\sigma_{yy}=\frac{e^{2}\tau }{\pi \hbar^{2}}E_{F}+\frac{m^{*}e^{2}\tau }{2\pi \hbar^{4}}\left[2\left(\alpha^{2}+\beta^{2}\right)-\left|\alpha^{2}-\beta^{2}\right|\right]$$

3b $$\sigma_{xy}=\sigma_{yx}=-\frac{m^{*}e^{2}\tau }{2\pi \hbar^{4}}\alpha \beta \frac{\left|\alpha^{2}-\beta^{2}\right|}{\max\left(\alpha^{2},\beta^{2}\right)}$$

The symmetric off-diagonal conductivity σ_*xy*_ = σ_*yx*_ ∝ αβ is nonzero only when both Rashba and Dresselhaus interactions are present, reflecting the lowering of the rotational symmetry by their coexistence. Equations 3a and 3b lead to the following expression of the angle dependence of the electrical resistivity (See Supplementary Note 1 for details):

4 $$\rho_{∥}=\rho_{0}-\Delta \rho \;\cos2\theta ,,\rho_{⊥}=\Delta \rho \;\sin2\theta$$

where ρ_∥_ (ρ_⊥_) is the longitudinal (transverse) resistivity, θ is the in-plane current angle with respect to [110], ρ_0_ = σ_*xx*_/(σ_*xx*_^2^ – σ_*xy*_^2^), and Δρ = ρ_0_ σ_*xy*_/σ_*xx*_. This result is consistent with Figure 2d and highlights the importance of the coexistence of Rashba and Dresselhaus interactions for the 2-fold angular dependence, since Δρ ∝ αβ.

The correspondence between the angular dependence of ρ_∥_ and that of the spin–orbit field in Figure 2f can also be understood within this formalism. From a transport perspective, this correspondence is related to current-induced spin polarization. In a RD system, an applied electric field generates a nonequilibrium spin density ⟨S⟩ = $\frac{\hbar }{2S}\underset{k}{\sum }\mathrm{Tr}\left[\sigma \rho_{k}^{\left(1\right)}\right]$ through the Edelstein effect. This spin polarization contributes to the charge current through the spin-dependent part of the velocity operator in $v_{k}$ = $\frac{\hbar k}{m^{*}}+\frac{\sigma_{x}}{\hbar }\left(\beta \mathrm{x̂}+\alpha ŷ\right)-\frac{\sigma_{y}}{\hbar }\left(\alpha \mathrm{x̂}+\beta ŷ\right)$ . Although this argument is heuristic, it captures the same angular dependence as the RD spin–orbit field |**d**(**k**)|^2^ = *k* ^2^ (α^2^ + β^2^ + 2αβ cos 2γ), where γ is the angle of **k** measured from the [110] direction. Thus, the anisotropic part of ρ_∥_ follows the same angular symmetry, δρ_∥_ (θ) ∝ αβ cos 2θ, and originates from the same αβ Rashba–Dresselhaus interference that modulates |**d**(**k**)|^2^, and hence shares its angular symmetry. We next examine the second-harmonic response. Figure 3a shows *E*_*xy*_^2ω^, measured at 300 K, as a function of current density for representative current orientations. For all directions, *E*_*xy*_^2ω^ exhibits a quadratic dependence on current density, confirming its second-order nonlinear origin. The magnitude strongly depends on the current angle: the response is maximal at θ = 0°, nearly vanishes at θ = 90°, and reverses sign between 45° and 135°, demonstrating pronounced crystallographic anisotropy of the nonlinear transverse transport. To compare the nonlinear response independently of the longitudinal conductivity, we extract the normalized figure of merit *E*_*xy*_^2ω^/(*E*_*xx*_^1ω^)^2^. As summarized in Figure 3b and 3c, the normalized signal exhibits cos^3^θ angular dependence, whose connection to the coexisting Rashba and Dresselhaus interactions is demonstrated below. The extracted magnitude reaches the order of 10^–9^ V^–1^ m. This value is comparable to those reported in representative nonlinear Hall systems, where bilayer WTe_2_^8^ exhibits values in the range of 6 × 10^–10^ to 1.4 × 10^–9^ V^–1^ m and topological-insulator Bi_2_Se_3_ surfaces^9^ show magnitudes between 3 × 10^–10^ and 8 × 10^–10^ V^–1^ m. The comparable order of magnitude demonstrates that sizable nonlinear Hall responses can be achieved even in conventional semiconductor quantum wells without relying on Berry-curvature-enhanced Weyl or topological band structures.

**Figure 3:**
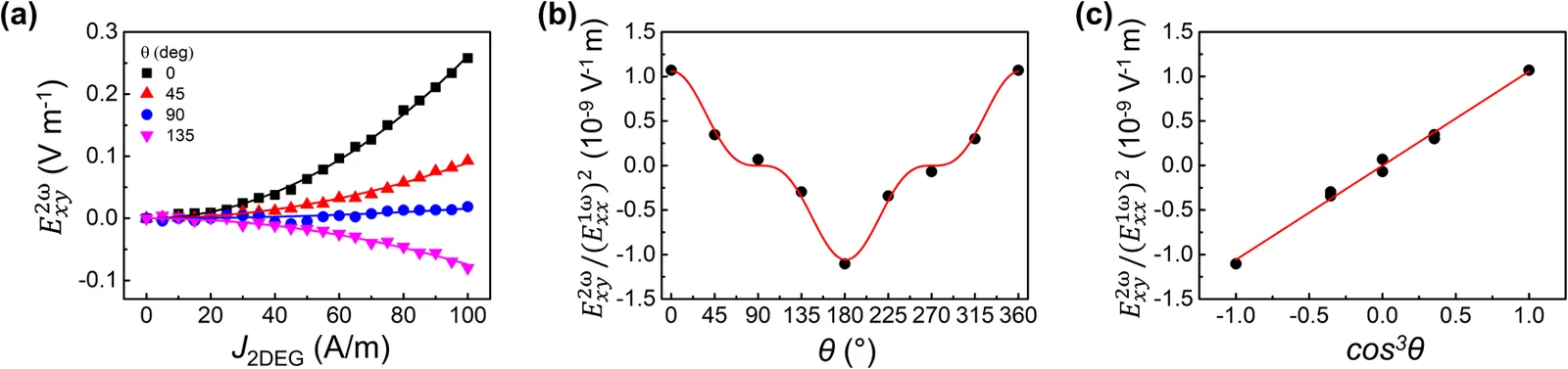
Angle-dependent nonlinear Hall response. (a) Second-harmonic transverse electric fields for representative current angles. The solid lines are quadratic fits to the data. (b) Normalized nonlinear Hall signal versus current angle. (c) Normalized nonlinear Hall response as a function of cos^3^ θ. The error bars in panels (b) and (c) are smaller than the size of the symbols. All measurements were carried out at 300 K.

With the current fixed along the [110] crystallographic direction, we measured the temperature dependence of *E*_*xy*_^2ω^ (Figure 4a). For a fixed current density, the nonlinear Hall signal increases with decreasing temperature. Since the carrier density remains nearly unchanged in our InAs quantum well while the mobility increases substantially at low temperature,^26^ this enhancement primarily reflects an increase in the conductivity rather than changes in carrier concentration. The quadratic dependence on current density is preserved at all temperatures, confirming the second-order transport origin of the signals. In Figures S2–S4, we show the nearly frequency-independent response, the consistent behavior under contact-polarity reversal, and the in-phase and quadrature components to assess possible artifacts.

**Figure 4:**
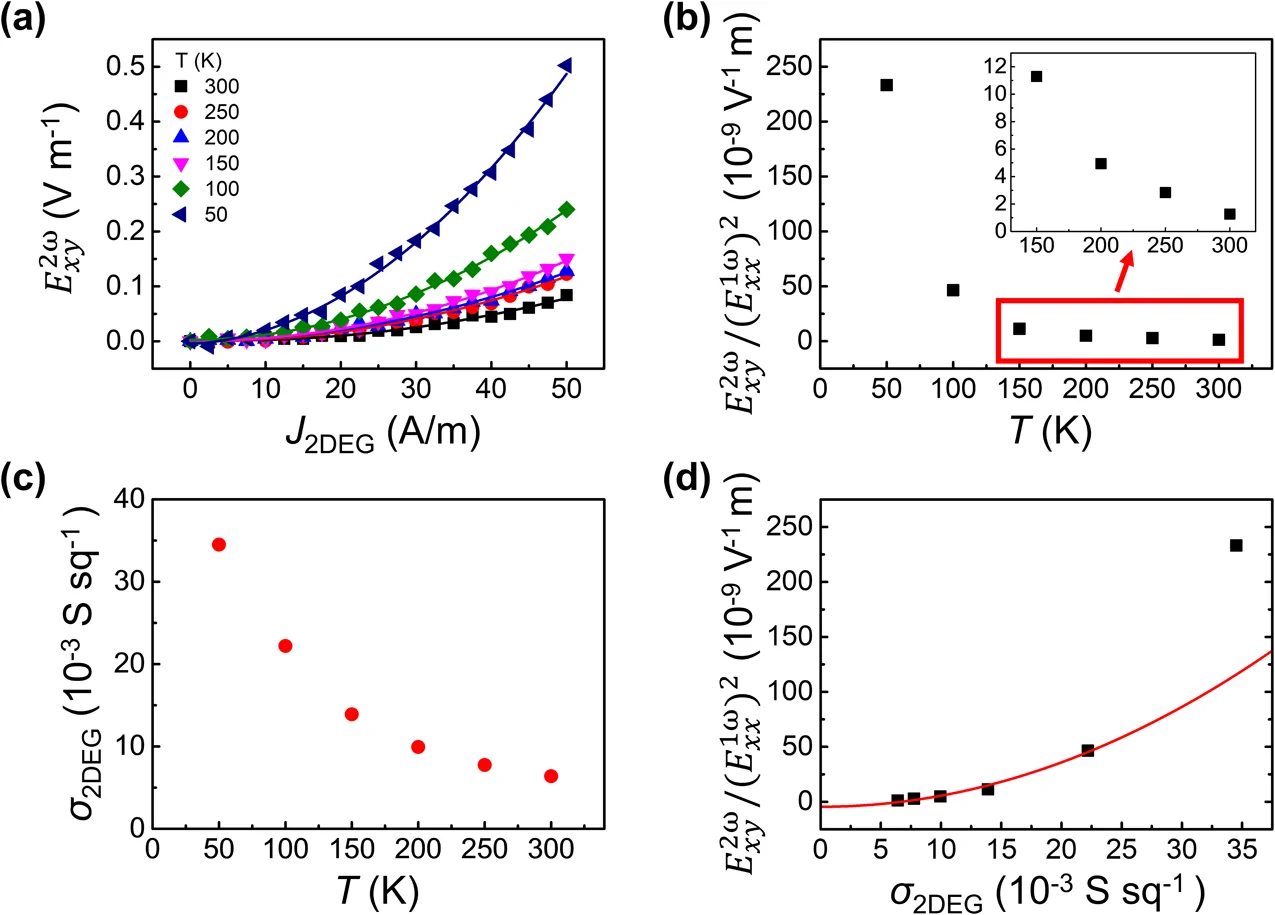
Temperature-dependent nonlinear Hall response. (a) Second-harmonic transverse electric fields at different temperatures. The solid lines are quadratic fits to the data. (b) Temperature dependence of the normalized nonlinear Hall signal. The inset shows an enlarged view of the high-temperature data. (c) Temperature dependence of the longitudinal conductivity σ_2DEG_. (d) Normalized nonlinear Hall signal as a function of σ_2DEG_ with quadratic fitting. The error bars in panel (d) are smaller than the size of the symbols. All measurements were performed with current along the [110] axis.

To further clarify the temperature dependence of the nonlinear response, we plot *E*_*xy*_^2ω^/(*E*_*xx*_^1ω^)^2^ as a function of temperature, as shown in Figure 4b. *E*_*xy*_^2ω^/(*E*_*xx*_^1ω^)^2^ increases significantly as the temperature decreases. For comparison, Figure 4c shows the temperature dependence of the 2DEG conductivity σ_2DEG_, which also increases markedly at low temperatures due to the strong enhancement of carrier mobility. The similar temperature trends suggest that the nonlinear Hall signal is closely related to carrier scattering.

To analyze the microscopic mechanism, we follow the established scaling approach for NHE systems and plot *E*_*xy*_^2ω^/(*E*_*xx*_^1ω^)^2^ as a function of σ_2DEG_, as shown in Figure 4d. The scaling relation is given by^7,8^

5 $$E_{xy}^{2\omega }/{\left(E_{xx}^{1\omega }\right)}^{2}=a\sigma_{2\mathrm{DEG}}^{2}+b$$

where *a* and *b* are material parameters, with *a* representing the skew-scattering contribution and *b* representing conductivity-independent contributions such as intrinsic and side-jump mechanisms.^4,12^ The conventional nonlinear Drude contribution ∝ σ_2DEG_ vanishes in a time-reversal-symmetric nonmagnetic system in the absence of a magnetic field.^4^ We excluded the data at 50 K following the previous reports that the quadratic conductivity dependence may break down in the high-conductivity regime. The deviation is possibly due to the increasing influence of static disorder, although its microscopic origin is still under debate.^12,15,27^ After fitting, the data are well-described by eq 5 with the fitting parameters *a* = 1.0 × 10^–4^ V^–1^ m S^–2^ and *b* = −4.5 × 10^–9^ V^–1^ m. Such scaling is commonly associated with skew-scattering–dominated transport, where the skew-scattering contribution increases more rapidly with conductivity and can therefore dominate the intrinsic or side-jump contributions (Figure S1). The observed behavior is therefore consistent with extrinsic scattering processes contributing to the nonlinear response in our system. This is also consistent with our minimal model, in which the intrinsic Berry-curvature-dipole contribution vanishes because the Berry curvature is proportional to **d**·(*∂*_*k*_*x*__ **d** × *∂*_*k*_*y*__ **d**), which is zero, and hence its dipole also vanishes.

To examine theoretically the nonlinear Hall response arising from the interplay between Rashba and Dresselhaus interactions and skew scattering, we first discuss the symmetry of the NHE. The observation of a nonzero *χ*_*yxx*_ implies the breaking of *C*_2_ symmetry. However, the Rashba–Dresselhaus Hamiltonian itself preserves *C*_2_ symmetry and therefore cannot by itself generate a nonzero *χ*_*yxx*_. The microscopic origin of the *C*_2_ symmetry breaking is difficult to identify unambiguously from the present measurements. As for possible origins, lattice mismatch and unintended strain^28^ may generate a finite polar component along a particular direction. Similar situations have been reported in nominally symmetric materials and structures,^29,30^ where residual or unintended symmetry breaking not apparent from the nominal structure was found to play an important role in the observed phenomena. The detailed origin of the symmetry breaking in our system goes beyond the scope of our work and requires further work.

A natural minimal representation of this *C*_2_ symmetry breaking is a finite polar component along a particular direction as the lowest angular harmonic term. In the presence of the lateral *C*_2_ symmetry breaking along a particular direction, the current-induced spin polarization corresponding to the lateral symmetry breaking may have a spin-*z* component to first order in the electric-field component that is perpendicular to the symmetry breaking axis.^31^ The anomalous Hall effect associated with this spin-*z* component can generate skew-scattering-induced NHE.

There are two remarks in this picture. First, in an ideal spin-polarized Rashba system with both chiral subbands occupied, the lowest-order skew-scattering contribution to the anomalous Hall effect vanishes,^32^ whereas finite skew-scattering contributions can arise beyond the lowest-order treatment,^33^ suggesting that the simple onefold contribution associated with this channel can be strongly suppressed. Second, upon adding a minimal symmetry-breaking term in the Rashba–Dresselhaus Hamiltonian, the momentum-resolved spin-*z* contribution contains a first-harmonic factor associated with the polar symmetry breaking and can acquire an additional 2-fold modulation through its dependence on the Rashba–Dresselhaus spin splitting |**d**(**k**)| (see Supplementary Note 2). Note that the 2-fold anisotropy in |d(k)| = $\sqrt{\left(\alpha^{2}+\beta^{2}\right)\left(k_{x}^{2}+k_{y}^{2}\right)+4\alpha \beta k_{x}k_{y}}$ is present only when both α and β are present, highlighting the importance of their coexistence. The combination of these angular harmonics may give rise to the cubic angular dependence observed in the experiment.

To explicitly demonstrate the above possibility, we implement this idea in Supplementary Note 2. As a result, we obtain a contribution to the second-order Hall current showing a cos^3^θ angular dependence.

6 $$j_{H}^{\left(2\right)}\propto E_{0}^{2}\lambda_{P}\alpha \beta \tau^{3}\;\cos^{3}\theta$$

where *j*_*H*_^(2)^ is the second-order Hall current flowing perpendicular to the electric field direction, *λ*_*P*_ is proportional to the strength of the polar *C*_2_-breaking contribution, θ is the angle of the electric field with respect to [110] and *E*_0_ is the amplitude of the ac electric field. Several remarks are noteworthy. First, the cos^3^θ dependence in eq 6 is in good agreement with the angular dependence shown in Figure 3b and 3c, consistent with the proposed scenario. However, because this demonstration assumes a particular direction of the *C*_2_-breaking term, the corresponding cubic contribution can generally take the form sin θ_0_ cos^3^θ + cos θ_0_ sin^3^ θ [eq (S15)], depending on this orientation, and eq 6 is recovered for θ_0_ = ±π/2. The observed phase θ_0_ = ±π/2 need not be accidental, but may reflect a correlation between the residual *C*_2_-breaking axis and a principal crystallographic axis of the Rashba–Dresselhaus Hamiltonian. Such a correlation could arise from anisotropic residual strain or other unintended symmetry breaking associated with the heterostructure, although its microscopic origin cannot be established from the present measurements. Further studies involving controlled strain or disorder engineering would help clarify this point. Second, the factor αβ indicates that the coexistence of Rashba and Dresselhaus interactions is required for obtaining a 2-fold modulation of spin splitting and the resulting cos^3^θ contribution. Third, the factor τ^3^ highlights the role of skew scattering. Without skew scattering, this τ^3^ scaling contribution to the nonlinear Hall conductivity is absent in our model (See Supplementary Note 2). This τ^3^ scaling is characteristic of the skew-scattering contribution to nonlinear Hall transport,^15^ leading to *E*_*xy*_^2ω^/(*E*_*xx*_^1ω^)^2^ ≈ σ_2DEG_^2^, as in eq 5. This is also consistent with our experimental result shown in Figure 4d. Fourth, although our demonstration is restricted to a specific setting, a similar physical route to a cubic angular dependence may apply more generally, involving the lowest-order polar component arising from unintended symmetry breaking and the 2-fold anisotropy of the spin splitting that can arise from the coexistence of the Rashba and Dresselhaus interactions.

In summary, we have demonstrated a sizable nonlinear Hall effect in an InAs quantum-well 2DEG with coexisting Rashba and Dresselhaus spin–orbit interactions. The second-harmonic transverse response exhibits clear quadratic current scaling and strong 2-fold angular anisotropy that directly tracks the effective spin–orbit field, establishing anisotropic spin–orbit coupling as the primary origin of the nonlinear transport. Conductivity-dependent scaling further indicates that skew scattering plays a dominant role in the observed response. Importantly, the nonlinear Hall signal persists up to room temperature and reaches magnitudes comparable to those of topological and Weyl materials. Our results show that complex band topology is not a prerequisite for achieving strong nonlinear transverse transport. Instead, conventional semiconductor 2DEGs provide a minimal, technologically compatible, and electrically tunable platform for investigating nonlinear Hall mechanisms. Our work may also motivate further exploration of gate-controlled spin–orbit engineering toward nonlinear Hall devices for rectification and frequency-doubling applications.

## Methods

### Material Growth and Device Fabrication

The InAs-based 2DEG heterostructure was grown on a semi-insulating InP (001) substrate by molecular beam epitaxy. The heterostructure consists of a 2-nm-thick InAs quantum well-embedded between In_0_._53_Ga_0_._47_As and In_0_._52_Al_0_._48_As barrier layers. An *n* ^+^-In_0_._52_Al_0_._48_As layer located beneath the quantum well was used as a carrier supply layer, resulting in structural inversion asymmetry along the growth direction. A thin InAs capping layer was deposited on the surface to protect the heterostructure during device fabrication.

Hall-bar devices were fabricated using standard photolithography followed by Ar-ion milling. The channel width and the separation between adjacent voltage probes were 25 μm. For angle-dependent measurements, Hall-bar devices were fabricated along four crystallographic directions corresponding to θ = 0°, 45°, 90°, and 135° with respect to the [110] crystal axis.

### Electrical Measurements

Electrical transport measurements were performed in a Physical Property Measurement System (PPMS). An alternating current was applied to the Hall-bar channel, and the first- and second-harmonic voltage signals were simultaneously detected using two SR860 lock-in amplifiers. The excitation frequency was fixed at 197.777 Hz, except for the frequency-dependent control measurements shown in Figure S2. Longitudinal and transverse voltages were measured for both harmonic components over a temperature range of 50–300 K. For nonlinear Hall measurements, the current density was defined as *J*_2DEG_ = *I*/*W*, where *I* is the applied current and *W* = 25 μm is the lithographically defined channel width. The electric fields were calculated by dividing the measured voltages by 25 μm, corresponding to both the channel width and the separation between adjacent voltage probes.

## Supplementary Material



## Notes

Supplementary Note 1, Derivation of the first-harmonic resistivity tensor; Supplementary Note 2, Derivation of the angular dependence of the second-harmonic Hall effect; Supplementary Figure 1, Separation of the skew-scattering contribution and the intrinsic and side-jump contributions; Supplementary Figure 2, Frequency dependence of the second-harmonic transverse response; Supplementary Figure 3, In-phase and quadrature components of the second-harmonic transverse response; Supplementary Figure 4, Second-harmonic transverse response under current and voltage contact-polarity reversals (PDF)
