# Supplementary material for: Nonlinear Hall Effect in a Semiconductor Heterostructure with Rashba and Dresselhaus Effects
Source: Nano Lett. 2026 Nov 2;26(44):15274–81. doi: 10.1021/acs.nanolett.6c03601 (PMC13761694; doi:10.1021/acs.nanolett.6c03601)
Supplement: Supplementary file 1 [file nl6c03601_si_001.pdf]

Supplementary Information

Nonlinear Hall Effect in a Semiconductor Heterostructure with Rashba and Dresselhaus Effects

Jeong Ung Ahn[1,2‡], Junyeong Choi[3,4‡], Minseong Kwon[2,5], Won-Young Choi[2,6], Ki Hyuk Han[2], Cheol Jun Kim[2], Chaun Jang[2], Dong-Soo Han[2], OukJae Lee[2], Byoung-Chul Min[2], Kyoung-Whan Kim[3,4*], and Hyun Cheol Koo[1,2*]

[1] KU-KIST Graduate School of Converging Science and Technology, Korea University, Seoul 02841, Republic of Korea

[2] Center for Semiconductor Technology, Korea Institute of Science and Technology, Seoul 02792, Republic of Korea

[3] Department of Physics, Yonsei University, Seoul 03722, Republic of Korea

[4] Center for Quantum Dynamics of Angular Momentum, Pohang University of Science and Technology, Pohang 37673, Republic of Korea

[5] Department of Physics, Kyung Hee University, Seoul 02447, Republic of Korea

[6] Department of Materials Science and Engineering, Seoul National University, Seoul 08826, Republic of Korea

* Corresponding authors: Kyoung-Whan Kim kwkim@yonsei.ac.kr, Hyun Cheol Koo hckoo@kist.re.kr

‡ These authors contributed equally to this work.

Note 1. Derivation of the first-harmonic resistivity tensor

We start from the quantum Liouville equation with the relaxation-time approximation as presented in the main text. After linearization with respect to the electric field, we obtain

$$\mathcal{L}_{\mathbf{k}}\left[\rho_{\mathbf{k}}^{(1)}\right] = -\frac{i}{\hbar}\left[H_{\mathbf{k}}, \rho_{\mathbf{k}}^{(1)}\right] - \frac{\rho_{\mathbf{k}}^{(1)}}{\tau} = -\frac{e\mathbf{E}}{\hbar}\cdot\mathcal{D}_{\mathbf{k}}\rho_{\mathbf{k}}^{(0)}, \tag{S1}$$

where $\mathcal{L}_{\mathbf{k}}[\cdots] = -\frac{i}{\hbar}[H_{\mathbf{k}}, \cdots] - \frac{1}{\tau}(\cdots)$ is the modified Liouville operator including the relaxation term. The first-order correction to the density matrix is then given by

$$\rho_{\mathbf{k}}^{(1)} = -\mathcal{L}_{\mathbf{k}}^{-1}\left[\frac{e\mathbf{E}}{\hbar}\cdot\mathcal{D}_{\mathbf{k}}\rho_{\mathbf{k}}^{(0)}\right]. \tag{S2}$$

It is useful to clarify the roles of the operators $\mathcal{L}_{\mathbf{k}}^{-1}$ and $\mathcal{D}_{\mathbf{k}}$. In the eigenstate basis $|n\mathbf{k}\rangle$ for $n = \pm$ with the energy eigenvalues $\mathcal{E}_{\mathbf{k}\pm} = \frac{\hbar^2 k^2}{2m^*} \pm |\mathbf{d}(\mathbf{k})|$,

$$-(\mathcal{L}_{\mathbf{k}}[B])_{mn} = \left(\frac{i}{\hbar}[H_{\mathbf{k}}, B] + \frac{1}{\tau}B\right)_{mn} = \frac{i}{\hbar}(\mathcal{E}_{\mathbf{k}m} - \mathcal{E}_{\mathbf{k}n})B_{mn} + \frac{1}{\tau}B_{mn}, \tag{S3}$$

for any 2×2 operator B. Provided that $\mathcal{E}_{\mathbf{k}+} - \mathcal{E}_{\mathbf{k}-} = 2|\mathbf{d}(\mathbf{k})| \gg \hbar/\tau$, the above equation is equivalent to

$$\mathcal{L}_{\mathbf{k}}^{-1}[B] = \begin{pmatrix} -\tau B_{++} & \dfrac{i\hbar}{2|\mathbf{d}(\mathbf{k})|}B_{+-} \\ -\dfrac{i\hbar}{2|\mathbf{d}(\mathbf{k})|}B_{-+} & -\tau B_{--} \end{pmatrix}. \tag{S4}$$

From $\rho_{\mathbf{k}}^{(0)} = f(H_{\mathbf{k}})$ where f is the Fermi-Dirac distribution, we obtain $\left[\mathcal{D}_{\mathbf{k}}\rho_{\mathbf{k}}^{(0)}\right]_{nm} = -(\partial_{\mathbf{k}}\mathcal{E}_{\mathbf{k}n})\delta(\mathcal{E}_{\mathbf{k}n} - E_F)\delta_{nm} - i\left[\mathbf{A}(\mathbf{k}), \rho_{\mathbf{k}}^{(0)}\right]_{nm}$ whose matrix representation in the eigenstate basis is given by

$$\mathcal{D}_{\mathbf{k}}\rho_{\mathbf{k}}^{(0)} = -\begin{pmatrix} (\partial_{\mathbf{k}}\mathcal{E}_{\mathbf{k}+})\delta(\mathcal{E}_{\mathbf{k}+} - \mathcal{E}_F) & -i[f(\mathcal{E}_{\mathbf{k}+}) - f(\mathcal{E}_{\mathbf{k}-})]\mathbf{A}_{+-}(\mathbf{k}) \\ -i[f(\mathcal{E}_{\mathbf{k}-}) - f(\mathcal{E}_{\mathbf{k}+})]\mathbf{A}_{-+}(\mathbf{k}) & (\partial_{\mathbf{k}}\mathcal{E}_{\mathbf{k}-})\delta(\mathcal{E}_{\mathbf{k}-} - \mathcal{E}_F) \end{pmatrix}. \tag{S5}$$

Using these matrix representations, the first-harmonic current can be calculated as

$$j_i^{(1)} = -\frac{e}{S}\sum_{\mathbf{k}}\mathrm{Tr}\left[v_{\mathbf{k}i}\rho_{\mathbf{k}}^{(1)}\right] = \frac{e^2 E_j}{S\hbar}\sum_{\mathbf{k}}\mathrm{Tr}\left[v_{\mathbf{k}i}\mathcal{L}_{\mathbf{k}}^{-1}\left[\mathcal{D}_j\rho_{\mathbf{k}}^{(0)}\right]\right]. \tag{S6}$$

Here and throughout this note, repeated indices are summed over, and we denote $\mathcal{D}_{k_j}$ and ∂_{k_j} by $\mathcal{D}_j$ and ∂_j, respectively, for notational simplicity. Substituting Eqs. (S4) and (S5) into Eq. (S6) gives

$$j_i^{(1)} = \frac{e^2 E_j}{S\hbar}\sum_{\mathbf{k}}\Bigg[\frac{\tau}{\hbar}(\partial_i \mathcal{E}_{\mathbf{k}+})(\partial_j \mathcal{E}_{\mathbf{k}+})\delta(\mathcal{E}_{\mathbf{k}+} - \mathcal{E}_F) + \frac{\tau}{\hbar}(\partial_i \mathcal{E}_{\mathbf{k}-})(\partial_j \mathcal{E}_{\mathbf{k}-})\delta(\mathcal{E}_{\mathbf{k}-} - \mathcal{E}_F) + 2[f(\mathcal{E}_{\mathbf{k}+}) - f(\mathcal{E}_{\mathbf{k}-})]\mathrm{Im}[A_{i+-}(\mathbf{k})A_{j-+}(\mathbf{k})]\Bigg]. \quad \text{(S7)}$$

In the clean limit, the last term is negligible compared with the other terms. To see this, we use $\mathrm{Im}[A_{i+-}(\mathbf{k})A_{j-+}(\mathbf{k})] \sim \frac{\hbar^2}{4|\mathbf{d}(\mathbf{k})|^2}\mathrm{Im}[v_{i+-}(\mathbf{k})v_{j-+}(\mathbf{k})]$ and expand $f(\mathcal{E}_{\mathbf{k}+}) - f(\mathcal{E}_{\mathbf{k}-}) \approx 2|\mathbf{d}(\mathbf{k})|f'(\mathcal{E}_{\mathbf{k}0})$ to leading order in the spin-orbit splitting, where $\mathcal{E}_{\mathbf{k}0} = \frac{\mathcal{E}_{\mathbf{k}+} + \mathcal{E}_{\mathbf{k}-}}{2}$. Then, the last term in Eq. (S7) is $\sim \hbar^2 \frac{f'(\mathcal{E}_{\mathbf{k}0})}{|\mathbf{d}(\mathbf{k})|}\mathrm{Im}[v_{i+-}(\mathbf{k})v_{j-+}(\mathbf{k})]$ which is much smaller than $\frac{\tau}{\hbar}(\partial_i \mathcal{E}_{\mathbf{k}n})(\partial_j \mathcal{E}_{\mathbf{k}n})\delta(\mathcal{E}_{\mathbf{k}n} - \mathcal{E}_F)$ in the limit of $2|\mathbf{d}(\mathbf{k})| \gg \hbar/\tau$. Finally, we obtain $j_i^{(1)} = \sigma_{ij}E_j$ where

$$\sigma_{ij} = \frac{e^2\tau}{S\hbar^2}\sum_{\mathbf{k}n}(\partial_i \mathcal{E}_{\mathbf{k}n})(\partial_j \mathcal{E}_{\mathbf{k}n})\delta(\mathcal{E}_{\mathbf{k}n} - E_F). \quad \text{(S8)}$$

This expression is consistent with the Kubo-Greenwood formula. The electrical conductivity for the Rashba-Dresselhaus model can be obtained by substituting the anisotropic band structure $\mathcal{E}_{\mathbf{k}\pm} = \frac{\hbar^2 k^2}{2m^*} \pm |\mathbf{d}(\mathbf{k})|$ with $|\mathbf{d}(\mathbf{k})| = |\mathbf{k}|\sqrt{\alpha^2 + \beta^2 + 2\alpha\beta\cos 2\gamma}$ where γ is the angle of the crystal momentum $\mathbf{k}$ with respect to [110]. After performing the straightforward but tedious $\mathbf{k}$-space integration in Eq. (S8), one obtains the analytic expressions for the first-harmonic conductivities σ_{xx}, σ_{xy}, and σ_{yy}, given in Eq. (3) of the main text.

To obtain the angular dependence, we consider an electric field applied at an angle θ with respect to the [110] direction. Taking $\hat{\mathbf{x}}' = \cos\left(\theta + \frac{\pi}{4}\right)\hat{\mathbf{x}} + \sin\left(\theta + \frac{\pi}{4}\right)\hat{\mathbf{y}}$ and $\hat{\mathbf{y}}' = \hat{\mathbf{z}} \times \hat{\mathbf{x}}'$, the transformed conductivity tensor in this rotated coordinate system is given by $\begin{pmatrix} \sigma_{xx} + \sigma_{xy}\cos 2\theta & -\sigma_{xy}\sin 2\theta \\ -\sigma_{xy}\sin 2\theta & \sigma_{xx} - \sigma_{xy}\cos 2\theta \end{pmatrix}$. Inverting this matrix, we obtain the following resistivity tensor.

$$\rho_{ij} = \frac{1}{\sigma_{xx}^2 - \sigma_{xy}^2}\begin{pmatrix} \sigma_{xx} - \sigma_{xy}\cos 2\theta & \sigma_{xy}\sin 2\theta \\ \sigma_{xy}\sin 2\theta & \sigma_{xx} + \sigma_{xy}\cos 2\theta \end{pmatrix}. \quad \text{(S9)}$$

The upper-left component gives $\rho_{\parallel}$, and the off-diagonal components give $\rho_{\perp}$, as presented in the main text.

Note 2. Derivation of the angular dependence of the second-harmonic Hall effect

(1) Role of skew scattering in the τ^3 nonlinear Hall response

To calculate the second-order response, we first consider the next-order solution of the quantum Liouville equation. For an electric field applied along a fixed *j*-direction, we obtain

$$\rho_{\mathbf{k}}^{(2)} = -\frac{eE_j}{\hbar}\mathcal{L}_{\mathbf{k}}^{-1}\left[\mathcal{D}_j\rho_{\mathbf{k}}^{(1)}\right] = \frac{e^2E_j^2}{\hbar^2}\mathcal{L}_{\mathbf{k}}^{-1}\mathcal{D}_j\mathcal{L}_{\mathbf{k}}^{-1}\mathcal{D}_j\left[\rho_{\mathbf{k}}^{(0)}\right]. \tag{S10}$$

Here no summation over *j* is implied in this section. However, the current obtained from Eq. (S10), $j_i^{(2)} = -\frac{e}{S}\sum_{\mathbf{k}}\mathrm{Tr}\left[v_{\mathbf{k}i}\rho_{\mathbf{k}}^{(2)}\right]$, contains only two factors of $\mathcal{L}_{\mathbf{k}}^{-1}$. Since $\mathcal{L}_{\mathbf{k}}^{-1} \propto \tau$ for the leading intraband response, this contribution alone cannot account for the experimentally observed τ^3 scaling. This indicates that an additional ingredient is required.

Motivated by the discussion in the main text, we consider a modified quantum Liouville equation including a skew-scattering term,

$$\frac{i}{\hbar}[H_{\mathbf{k}},\rho_{\mathbf{k}}] - \frac{e\mathbf{E}}{\hbar}\cdot\mathcal{D}_{\mathbf{k}}\rho_{\mathbf{k}} = -\frac{\rho_{\mathbf{k}}-\rho_{\mathbf{k}}^{(0)}}{\tau} - \mathcal{W}_{\mathbf{k}}[\rho_{\mathbf{k}}], \qquad \mathcal{W}_{\mathbf{k}}[\rho_{\mathbf{k}}] = \frac{1}{2}\sum_{\mathbf{k}'}\{\eta_{\mathbf{k}\mathbf{k}'},\rho_{\mathbf{k}'}\}. \tag{S11}$$

Here, $\mathcal{W}_{\mathbf{k}}$ denotes the skew-scattering operator satisfying $\eta_{\mathbf{k}\mathbf{k}'} = -\eta_{\mathbf{k}'\mathbf{k}}$ and we use an effective skew-scattering description consistent with the same spin-independent short-range delta-function impurity model used for the first-order response. The iterative solution is obtained by replacing each factor $-\mathcal{L}_{\mathbf{k}}^{-1}$ in Eq. (S10) by $(-\mathcal{L}_{\mathbf{k}}+\mathcal{W}_{\mathbf{k}})^{-1}$. To first order in $\mathcal{W}_{\mathbf{k}}$, we use $(-\mathcal{L}_{\mathbf{k}}+\mathcal{W}_{\mathbf{k}})^{-1} \approx -\mathcal{L}_{\mathbf{k}}^{-1} - \mathcal{L}_{\mathbf{k}}^{-1}\mathcal{W}_{\mathbf{k}}\mathcal{L}_{\mathbf{k}}^{-1}$. This gives

$$\begin{aligned}\rho_{\mathbf{k}}^{(2)} &= -\frac{eE_j}{\hbar}(1+\mathcal{L}_{\mathbf{k}}^{-1}\mathcal{W}_{\mathbf{k}})\mathcal{L}_{\mathbf{k}}^{-1}\left[\mathcal{D}_j\rho_{\mathbf{k}}^{(1)}\right] \\ &= \frac{e^2E_j^2}{\hbar^2}\mathcal{L}_{\mathbf{k}}^{-1}\mathcal{D}_j\mathcal{L}_{\mathbf{k}}^{-1}\mathcal{D}_j\left[\rho_{\mathbf{k}}^{(0)}\right] + \frac{e^2E_j^2}{\hbar^2}\mathcal{L}_{\mathbf{k}}^{-1}\mathcal{W}_{\mathbf{k}}\mathcal{L}_{\mathbf{k}}^{-1}\mathcal{D}_j\mathcal{L}_{\mathbf{k}}^{-1}\left[\mathcal{D}_j\rho_{\mathbf{k}}^{(0)}\right] \\ &\quad + \frac{e^2E_j^2}{\hbar^2}\mathcal{L}_{\mathbf{k}}^{-1}\mathcal{D}_j\mathcal{L}_{\mathbf{k}}^{-1}\mathcal{W}_{\mathbf{k}}\mathcal{L}_{\mathbf{k}}^{-1}\left[\mathcal{D}_j\rho_{\mathbf{k}}^{(0)}\right].\end{aligned} \tag{S12}$$

The last two terms contain three inverse Liouville operators and can therefore generate τ^3-scaling contributions. In the clean limit, the τ^3-scaling terms give the leading-order contribution. We therefore approximate $\mathcal{L}_{\mathbf{k}}^{-1}$ as $-\tau P_{\mathrm{diag}}$ where P_{diag} projects an operator onto its band-diagonal components in the eigenstate basis, as follows from Eq. (S4).

(2) Angular dependence of the second-harmonic Hall effect

Following the scenario stated in the main text, we assume that C_2 symmetry is broken by an unintended in-plane polar direction $\hat{\mathbf{P}}$, characterized by an angular phase offset θ_0. In the presence of an electric field along the θ direction, the first-order drift is given by $\delta f^{(1)}(\gamma) \propto \tau E_0 \cos(\gamma - \theta)$, where γ is the angle of the $\mathbf{k}$ vector and τ is the relaxation time. Hereafter, all angles are measured with respect to the [110] direction. More explicitly, $\mathbf{E} = E_0(\cos(\theta + \pi/4), \sin(\theta + \pi/4), 0)$, $\mathbf{k} = k(\cos(\gamma + \pi/4), \sin(\gamma + \pi/4), 0)$ and $\hat{\mathbf{P}} = (\cos(\theta_0 + \pi/4), \sin(\theta_0 + \pi/4), 0)$.

A possible minimal time-reversal-symmetric spin-orbit coupling consistent with this polar asymmetry is $H_P = \lambda_P \sigma_z (\hat{\mathbf{z}} \times \hat{\mathbf{P}}) \cdot \mathbf{k} = \lambda_P k \sin(\gamma - \theta_0)\, \sigma_z$. With this additional term, to first order in λ_P, the momentum-resolved spin-z component is given by $s_{z,\pm}^{(0)}(\gamma) = \pm \hbar \lambda_P k \sin(\gamma - \theta_0)/2|\mathbf{d}(\mathbf{k})|$, where $\pm$ denotes the spin band index. Combining this with $\delta f^{(1)}(\gamma)$ gives the leading momentum-resolved nonequilibrium out-of-plane spin polarization,

$$\delta s_z^{(1)}(\gamma) \propto \lambda_P \tau E_0 \sin(\gamma - \theta_0) \cos(\gamma - \theta)\, \mathcal{F}[|\mathbf{d}(\mathbf{k})|], \tag{S13}$$

where $\mathcal{F}$ collectively represents the effects of band occupation and velocity factors, band-dependent relaxation processes, energy-dependent Fermi wave vectors, vertex corrections, and the summation over the spin-split bands. For the present phenomenological demonstration, we model its angular dependence through $|\mathbf{d}(\mathbf{k})|$. Accordingly, $\mathcal{F}[|\mathbf{d}(\mathbf{k})|] = \sum_n \mathcal{F}_{2n} \cos 2n\gamma = \mathcal{F}_0 + \mathcal{F}_2 \cos 2\gamma + \mathcal{F}_4 \cos 4\gamma + \cdots$ where the angular anisotropy terms ($n \geq 1$) originate from the coexistence of the Rashba and Dresselhaus interactions as implied by the Rashba-Dresselhaus interference term in $|\mathbf{d}(\mathbf{k})| = \sqrt{(\alpha^2 + \beta^2)(k_x^2 + k_y^2) + 4\alpha\beta k_x k_y}$. As a side remark, for the isotropic component $\mathcal{F}_0$, integrating Eq. (S13) over the $\mathbf{k}$ direction gives $s_{z,\mathrm{tot}}^{(1)} \propto \lambda_P \tau E_0 \sin(\theta - \theta_0)$, which is consistent with the symmetry of the current-induced out-of-plane spin polarization driven by lateral symmetry breaking. Nevertheless, the $\mathbf{k}$ dependence should be retained until the final step in our calculation.

To minimize the mathematical complexity of the second-order response [Eq. (S12)], we focus on the angular dependence in $\mathcal{L}_{\mathbf{k}}^{-1} \mathcal{W}_{\mathbf{k}} \mathcal{L}_{\mathbf{k}}^{-1} \left[\mathcal{D}_j \rho_{\mathbf{k}}^{(1)}\right]$. We need to consider an additional vertex along the electric-field direction $[\mathcal{D}_j \to E \cos(\gamma' - \theta)]$, the skew vertex $[\mathcal{W}_{\mathbf{k}} \to \sin(\gamma - \gamma')]$, and two additional factors of τ corresponding to two $\mathcal{L}_{\mathbf{k}}^{-1}$ operators. Also, since the second-order Hall

contribution is proportional to $\mathrm{Tr}\left[v_{\mathrm{H}}\rho_{\mathbf{k}}^{(\mathbf{2})}\right]$, where v_{H} is the velocity operator along the Hall direction, we need to consider the transverse velocity vertex $[v_H \to \sin(\gamma-\theta)]$. Here γ and γ' are the outgoing and incoming momentum directions, respectively, and the skew vertex $\sin(\gamma-\gamma')$ is modelled as the lowest angular component of the antisymmetric part of the impurity vertex with respect to the exchange of incoming and outgoing momenta.

Collecting these factors, we obtain

$$j_H^{(2)} \propto \iint d\gamma d\gamma' \, E_0\tau^2 \sin(\gamma-\theta)\sin(\gamma-\gamma')\cos(\gamma'-\theta)\,\delta s_z^{(1)}(\gamma'), \tag{S14}$$

which generally depends on the coefficients $\mathcal{F}_{2n}$. The isotropic contribution $\mathcal{F}_0$ is discarded here, assuming that this onefold channel is strongly suppressed for the reason discussed in the main text. The next angular term arises from $\mathcal{F}_4$, which is of higher order in the Rashba-Dresselhaus anisotropy and is therefore neglected. The $\mathcal{F}_{2n}$ terms for $n \geq 3$ do not contribute to the integral. Therefore, retaining the $\mathcal{F}_2$ contribution, the right-hand side of Eq. (S14) becomes

$$-\frac{\pi^2}{2}\,\mathcal{F}_2 E_0^2 \lambda_P \tau^3 (\sin\theta_0 \cos^3\theta + \cos\theta_0 \sin^3\theta). \tag{S15}$$

Taking $\theta_0 = \pm\pi/2$ and recalling $\mathcal{F}_2 \propto \alpha\beta$ reproduces Eq. (6) in the main text. The detailed form depends on the specific value of θ_0. But, as we mention in the main text, we suspect that the direction of the unintended polar component arising during fabrication is correlated with a principal axis of the bare Hamiltonian. If this is indeed the case, the corresponding phase offsets would naturally be $\theta_0 = \pm\pi/2, 0, \pi$, depending on the orientation and polarity of the C_2-breaking component.

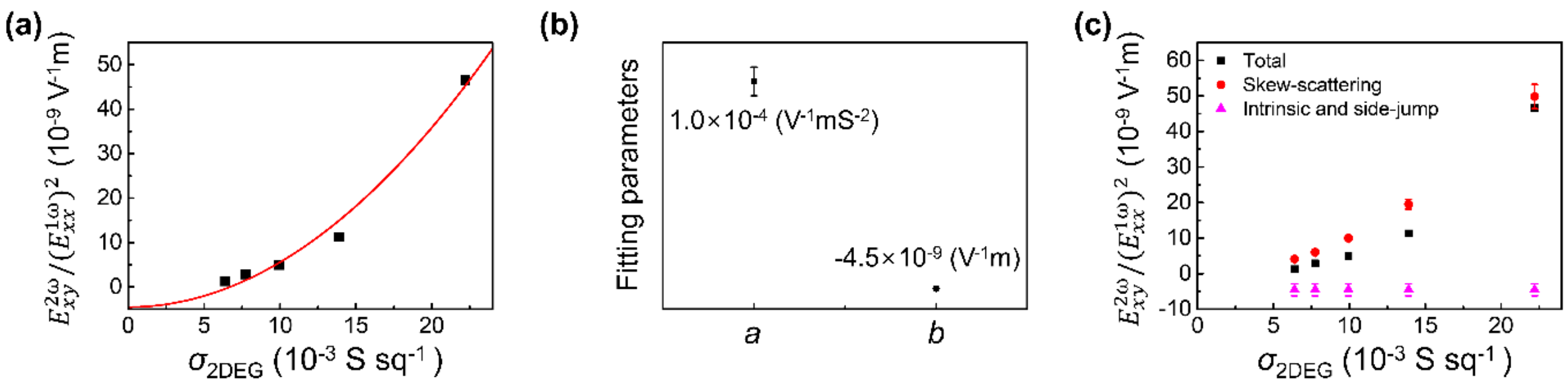

Figure S1. Separation of the skew-scattering contribution and the intrinsic and side-jump contributions. (a) Normalized nonlinear Hall signal $E_{xy}^{2\omega}/(E_{xx}^{1\omega})^2$ as a function of the longitudinal conductivity σ_{2DEG}. The solid line represents a fit to $E_{xy}^{2\omega}/(E_{xx}^{1\omega})^2 = a\sigma_{2DEG}^2 + b$. The fit uses data from 100 to 300 K. **(b)** Extracted fitting parameters a and b. **(c)** Comparison of the measured total signal with the skew-scattering contribution ($a\sigma_{2DEG}^2$) and the combined intrinsic and side-jump contribution (b), estimated from the fit. All measurements were performed with current along [110].

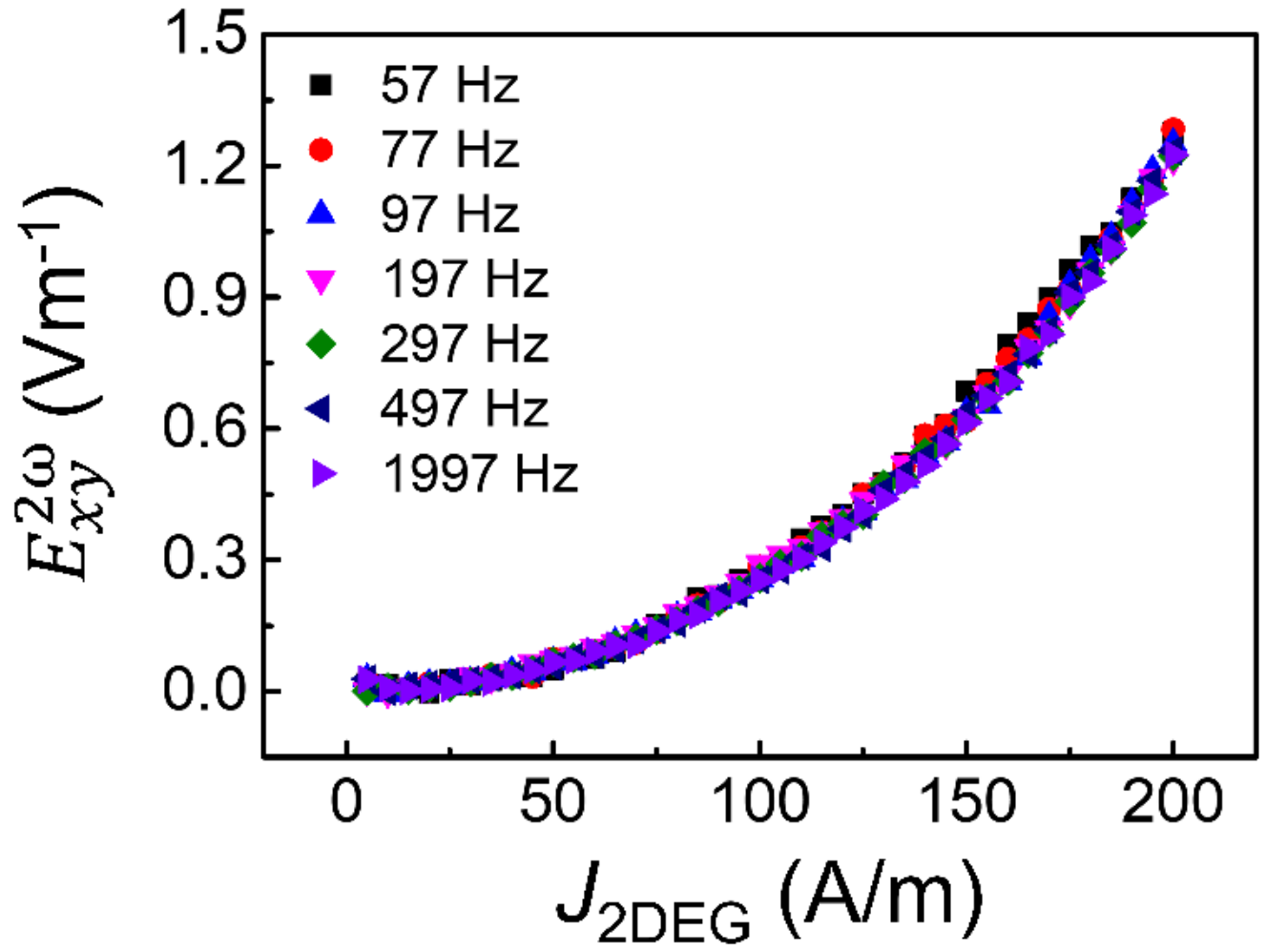

Figure S2. Frequency dependence of the second-harmonic transverse response. Second-harmonic transverse electric field $E_{xy}^{2\omega}$ as a function of current density J_{2DEG} at AC excitation frequencies ranging from 57 to 1,997 Hz. The frequencies indicated in the legend refer to the applied current, with the second-harmonic response detected at twice the excitation frequency. All measurements were performed at 300 K with current along the [110] axis.

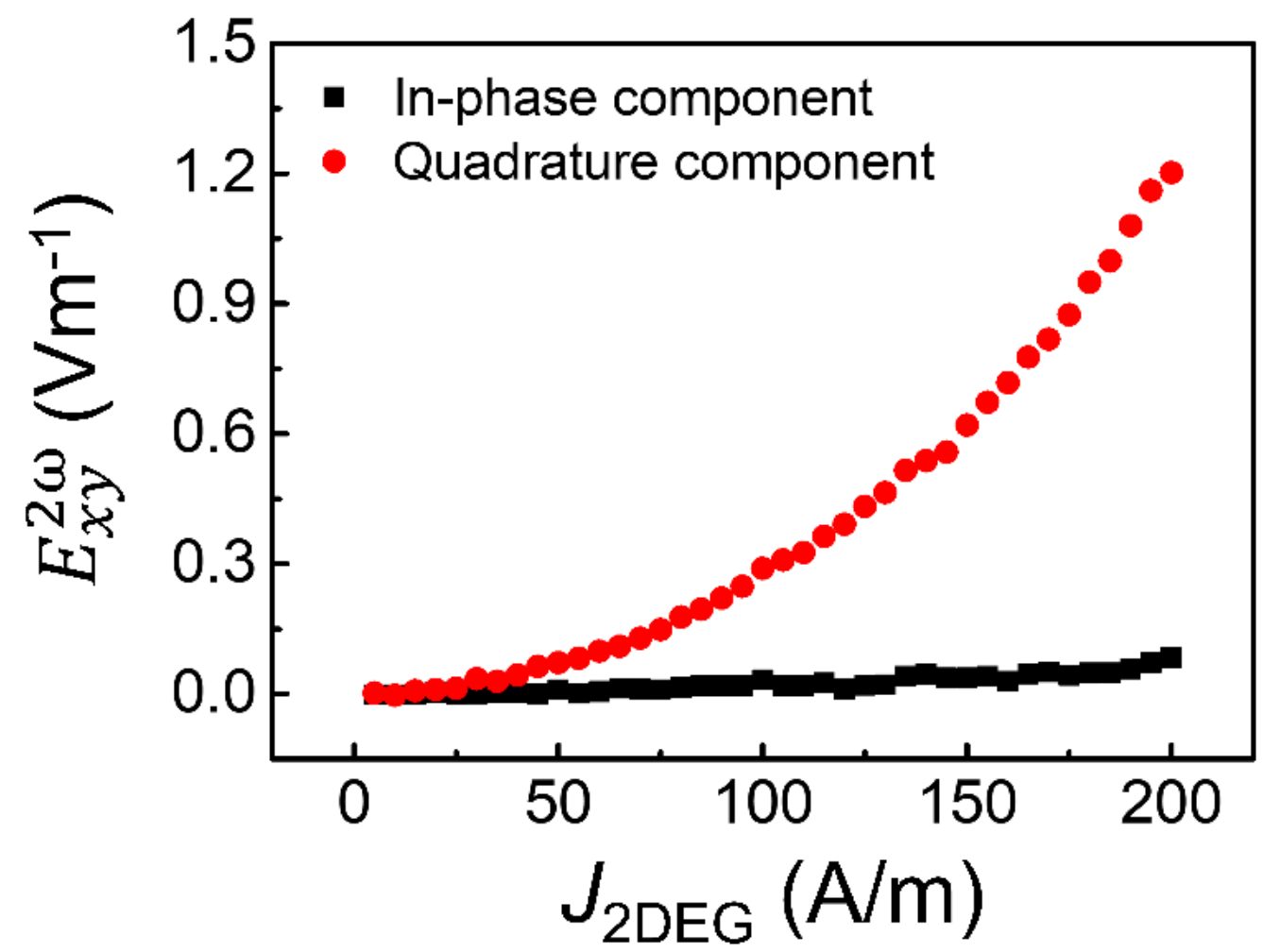

Figure S3. In-phase and quadrature components of the second-harmonic transverse response. In-phase and quadrature components of $E_{xy}^{2\omega}$ as a function of current density J_{2DEG}, measured at a representative frequency of 197 Hz. All measurements were performed at 300 K with current along the [110] axis.

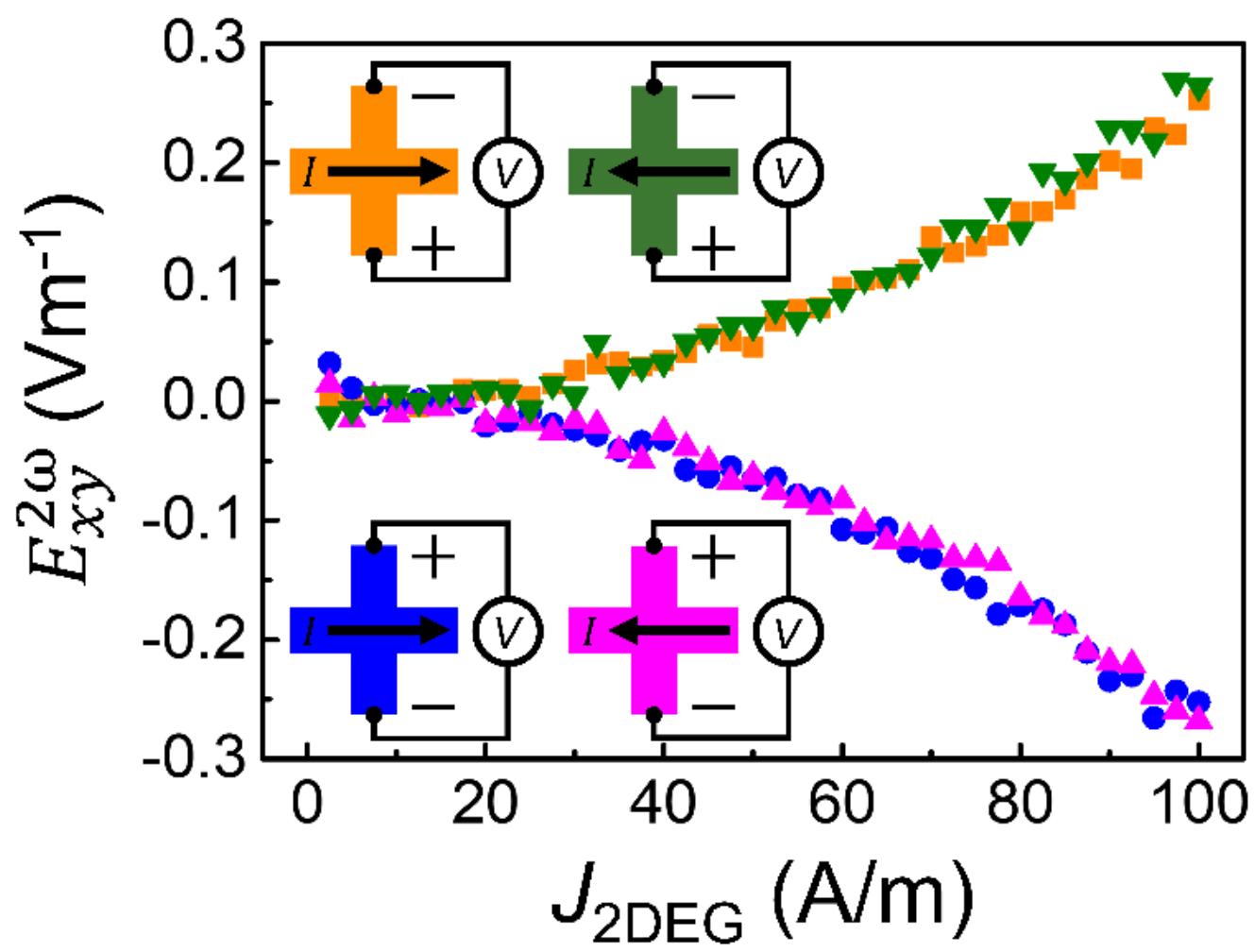

Figure S4. Second-harmonic transverse response under current and voltage contact-polarity reversals. $E_{xy}^{2\omega}$ as a function of current density J_{2DEG} measured under four combinations of current and voltage contact polarities. The inset schematics indicate the current direction and voltage-detection polarity for each configuration, with colors corresponding to the plotted data. All measurements were performed at 300 K with current along the [110] axis.
